# Regulation of the IGFBP-5 and MMP-13 genes by the microRNAs miR-140 and miR-27a in human osteoarthritic chondrocytes

**DOI:** 10.1186/1471-2474-10-148

**Published:** 2009-11-30

**Authors:** Ginette Tardif, David Hum, Jean-Pierre Pelletier, Nicolas Duval, Johanne Martel-Pelletier

**Affiliations:** 1 Osteoarthritis Research Unit, University of Montreal Hospital Research Centre (CRCHUM), Notre-Dame Hospital, Montreal, Quebec H2L 4M1, Canada; 2 Duval Clinique Orthopédique, Le Pavillon des Charmilles, 1487 Boulevard des Laurentides, Laval, Quebec H7M 2Y3, Canada

## Abstract

**Background:**

MMP-13 and IGFBP-5 are important factors involved in osteoarthritis (OA). We investigated whether two highly predicted microRNAs (miRNAs), miR-140 and miR-27a, regulate these two genes in human OA chondrocytes.

**Methods:**

Gene expression was determined by real-time PCR. The effect of each miRNA on IGFBP-5 and MMP-13 expression/production was evaluated by transiently transfecting their precursors (pre-miRNAs) and inhibitors (anti-miRNAs) into human OA chondrocytes. Modulation of IGFBP-5, miR-140 and miR-27a expression was determined upon treatment of OA chondrocytes with cytokines and growth factors.

**Results:**

IGFBP-5 was expressed in human chondrocytes with its level significantly lower (p < 0.04) in OA. Five computational algorithms identified miR-140 and miR-27a as possible regulators of MMP-13 and IGFBP-5 expression. Data showed that both miRNAs were expressed in chondrocytes. There was a significant reduction (77%, p < 0.01) in miR-140 expression in OA compared to the normal chondrocytes, whereas miR-27a expression was only slightly decreased (23%). Transfection with pre-miR-140 significantly decreased (p = 0.0002) and with anti-miR-140 significantly increased (p = 0.05) IGFBP-5 expression at 24 hours, while pre-miR-27a did not affect either MMP-13 or IGFBP-5. Treatment with anti-miR-27a, but not with anti-miR-140, significantly increased the expression of both MMP-13 (p < 0.05) and IGFBP-5 (p < 0.01) after 72 hours of incubation. MMP-13 and IGFBP-5 protein production followed the same pattern as their expression profile. These data suggest that IGFBP-5 is a direct target of miR-140, whereas miR-27a down-regulates, likely indirectly, both MMP-13 and IGFBP-5.

**Conclusion:**

This study is the first to show the regulation of these miRNAs in human OA chondrocytes. Their effect on two genes involved in OA pathophysiology adds another level of complexity to gene regulation, which could open up novel avenues in OA therapeutic strategies.

## Background

Many factors contribute to the overall degradation of cartilage observed in osteoarthritis (OA), either directly or indirectly by modulating anabolic factors. Examples of such molecules are the matrix metalloprotease (MMP)-13 and the insulin-like growth factor binding protein (IGFBP)-5. MMP-13 is a well known key player in cartilage biology and OA pathology because of its capacity to degrade, in addition to collagens, a wide range of matrix components [1-6]. Although a large number of factors including pro-inflammatory cytokines, growth factors, and fibronectin fragments have been reported to regulate MMP-13 expression [5,7,8], further knowledge about its regulation is needed in order to identify factors that could specifically inhibit this MMP while sparing others and, as such, avoid the unwanted side effects observed with broad spectrum MMP inhibitors [9,10]. IGFBPs are proteins known to modulate the availability/activity of the anabolic factor IGF-1. Evidence has shown that in the joint, IGFBP-5 plays an important storage role for IGF-1 [11]. Furthermore, results from a study using an OA dog model demonstrated that increasing IGFBP-5 concentration led to an increased level of IGF-1 and was associated with a reduction in cartilage destruction [12]. Despite its regulatory role in cartilage, the regulation of human IGFBP-5 itself has not yet been investigated in this tissue or in chondrocytes.

Although MMP-13 promoter regulation has been the subject of many publications [13-16], there is no report on the role of 3'-untranslated regions (3'-UTRs) on either its regulation or that of IGFBP-5. Since microRNAs (miRNAs) act on this region and are important regulators of gene expression, we investigated whether MMP-13 and IGFBP-5 are the targets of specific miRNAs.

miRNAs are small non-coding RNAs (20-25 nucleotides) naturally produced by the cells. They are derived from primary miRNA transcripts (70-100 nucleotides) that are processed in the nucleus to precursor miRNAs (pre-miRNAs) by the ribonuclease Drosha [17]. The pre-miRNAs are then transported into the cytoplasm where they are further processed into miRNAs by the ribonuclease Dicer [18]. The miRNAs play a role in gene silencing by regulating the stability or translational efficiency of target messenger RNA (mRNA). Depending on the degree of base pairing between the miRNA and the target mRNAs, the miRNAs either repress translation (imperfect pairing) or cleave the mRNAs (perfect pairing) [19]. Pairing generally occurs in the 3'UTR of the mRNAs. Another mechanism of miRNA-mediated mRNA degradation may involve AU-rich elements (AREs), which are located in the 3'-UTR of unstable mRNAs [20].

Several hundred miRNAs have been identified so far and initial studies have linked specific miRNAs to different tissues, developmental processes, and pathologies such as cancer [21-23]. Although algorithms are used to predict potential mRNA targets, only a few miRNAs have been validated and assigned to specific mRNAs.

The cellular outcomes of miRNA-mediated gene regulation are complex, as some miRNAs decrease while others increase cell growth, and still others increase the level of apoptosis [22]. However, because of their role, miRNAs may represent another avenue for therapeutic intervention in arthritic diseases.

The importance of miRNAs in joint pathologies and in inflammatory events has been addressed only recently. Stanczyk et al [24] reported that the expression of miR-155 and miR-146a was increased in synovial fibroblasts from rheumatoid arthritis (RA) patients as compared to OA. The miR-146 was also found to be up-regulated in peripheral blood mononuclear cells [25] and in synovial tissues [26] from RA patients. Moreover, the expression of miR-146 and miR-155 was also shown to be up-regulated by bacterial endotoxins and the pro-inflammatory cytokines interleukin-1β (IL-1β) and tumor necrosis factor-α (TNF-α) [27]. In a recent study [28], miR-146a was reported to be expressed mostly in OA cartilage showing mild scores and its expression was stimulated in normal chondrocytes by IL-1β. These findings thus suggest that some miRNAs could be of importance in the inflammatory events of arthritis.

There have been few reports on the role of miRNAs in cartilage biology. Tuddenham et al [29] reported the presence of miR-140 in cartilaginous tissues of the developing mouse and showed that this miRNA targeted the mouse histone deacetylase 4 mRNA. Kobayashi et al [30] showed that Dicer, an enzyme involved in the miRNA pathway, was essential for chondrocyte function in mice; the growth plates from Dicer-null mice demonstrated a progressive reduction in the proliferating pool of chondrocytes, leading to severe skeletal growth defects and premature death of the mice. The miR-199* was also recently shown to control chondrogenesis in mice, via direct targeting to Smad1 [31]. In humans, there is to our knowledge only one study in which the miRNAs have been profiled comparing normal and OA cartilage [32]. In this study it was found that 16 miRNAs were differentially expressed when OA was compared to normal cartilage. Moreover, comparison with clinical data revealed that some miRNAs (miR-22 and miR-33) and proteins, peroxisome proliferator-activated receptor-α (PPARα), bone morphogenic protein-7 (BMP-7) and IL-1β, highly correlate with body mass index. Moreover, as mentioned above, miR-146a has also been found expressed in a subset of OA cartilage [28].

We thus investigated whether MMP-13 and IGFBP-5 are regulated by miRNAs in human OA chondrocytes. We identified the miRNAs miR-140 and miR-27a as regulators of these two genes and studied their expression and regulation in normal and OA human chondrocytes. This study provides a more comprehensive understanding of the overall regulation of MMP-13 and IGFBP-5.

## Methods

### Specimen selection

Human cartilage was obtained from femoral condyles and tibial plateaus. Normal (control) cartilage was obtained from individuals within 12 hours of death (mean age ± SEM: 57 ± 8 years). These individuals had no history of joint disease and died of causes unrelated to arthritic diseases. The tissues were examined macroscopically and microscopically to ensure that only normal tissue was used. Human OA cartilage was obtained from patients undergoing total knee arthroplasty (72 ± 2 years). All patients had been evaluated by a certified rheumatologist and diagnosed as having OA according to the American College of Rheumatology criteria [33]. These specimens represented moderate to severe OA [34]. At the time of surgery, the patients had symptomatic disease requiring medical treatment. None had received intra-articular steroid injections within three months prior to surgery. The Institutional Ethics Committee Board of the Notre-Dame Hospital approved the use of the human articular tissues and patients signed informed consent.

### Cell culture

Chondrocytes were released from cartilage by sequential enzymatic digestion at 37°C, as previously described [5,8]. The cells were seeded at high density (10^5^/cm^2^) and cultured in Dulbecco's modified Eagle's medium (DMEM; Gibco BRL, Burlington, ON, Canada) supplemented with 10% heat-inactivated fetal calf serum (FCS; Gibco BRL) and an antibiotic mixture (100 units/ml penicillin base and 100 μg/ml streptomycin base; Gibco BRL) at 37°C in a humidified atmosphere. Primary chondrocytes were used when comparing expression levels in normal and OA chondrocytes; first-passage cultured chondrocytes were used in the other experiments.

The effects of cytokines and growth factors on IGFBP-5 and miRNA expression levels were assessed by pre-incubating confluent chondrocytes in DMEM/0.5% FCS for 20 hours containing the following factors: IL-1β (100 pg/ml), TNF-α (5 ng/ml), interferon-γ (IFN-γ, 10 U/ml), IL-4 (10 ng/ml), IL-10 (10 ng/ml), transforming growth factor-β (TGF-β, 10 ng/ml), BMP-2 (10 ng/ml), and epidermal growth factor (EGF, 10 ng/ml). The expression levels of IGFBP-5, miR-140, and miR-27a were quantified by real-time polymerase chain reaction (PCR).

### Total RNA extraction and real-time PCR

Total RNA was extracted, quantified and treated with DNase as described previously [35]. Real time PCR was performed in the Rotor-Gene RG-3000A (Corbett Research, Mortlake, Australia) with the SYBR Green PCR Master Mix (Qiagen, Valencia, CA, USA). The PCR parameters were as described [35]. The data were given as a threshold cycle (C_t_). Fold changes in gene expression were calculated as 2^-Δ(ΔCt)^. The primer efficiencies for the genes were the same as those for the housekeeping gene GAPDH, the expression level of which was used to normalize the results and assigned an arbitrary value of 1. The sequences of the human specific primers were 5'-CAGAACATCATCCCTGCCTCT(S) and 5'-GCTTGACAAAGTGGTCGTTGAG(AS) for GAPDH, 5'-CTTAGAGGTGACTGGCAAAC(S) and 5'-GCCCATCAAATGGGTAGAAG(AS) for MMP-13, 5'-TGAAGCAGTGAAGAAGGAC(S) and 5'-CTGCTTTCTCTTGTAGAATC(AS) for IGFBP-5, 5'-GAGATGCCTTCAGCAGAGTG(S) and 5'-ACATGCGCCTTGATGTCTG(AS) for IL-10, 5'-CTCACATCAAGCTACAACTTC(S) and 5'-GGTCCTGTTTTGGATCCAAG(AS) for basic fibroblast growth factor (bFGF).

### Extraction and quantification of miRNAs

miRNA was extracted with the mirVana kit (Ambion, Austin, TX, USA). This kit is designed especially for the isolation of small RNAs by enriching the population of RNAs that are 200 bases and smaller. The extracted RNA was reverse-transcribed with the TaqMan MicroRNA Reverse Transcription kit (Applied Biosystems, Foster City, CA, USA); the miRNAs miR-140 and miR-27a were quantified with TaqMan MicroRNA Assays specific for each mature miRNA (Applied Biosystems). Normalization of the miRNA expression data was done with the endogenous control gene RNU24 (small nucleolar RNU24), the expression of which has been shown to be high with little variation across tissue samples; it was assigned an arbitrary value of 1. The control gene was processed similarly to the miRNAs by extraction with the mirVana kit followed by the specific TaqMan microRNA assay.

### Transfection of pre-miR and anti-miR molecules

OA chondrocytes were transfected with pre-miRNA and anti-miRNA molecules specifically targeting the human miR-140 and miR-27a (45 nM final concentration; Applied Biosystems) in DMEM and the HiPerfect Transfection Reagent (3% final concentration; Qiagen). Fluorescent Cy™3-labeled double-stranded RNA oligonucleotides (45 nM final concentration) were used to monitor transfection efficiency. This protocol routinely results in a chondrocyte transfection efficiency of more than 90%. Cells, either non-transfected or transfected with non-targeting (random) pre-miRNA or anti-miRNA, served as controls and provided similar results.

### Protein production

The culture media were used for quantification of protein production. MMP-13 protein levels were quantified with the Fluorokine MAP Human MMP-13 kit (sensitivity 82 pg/ml; R&D Systems, Minneapolis, MN, USA) using the LiquiChip Luminex apparatus (Qiagen). IGFBP-5 protein levels were measured using the DuoSet ELISA Development kit (sensitivity 300 pg/ml; R&D Systems).

### Statistical analysis

Values are expressed as mean ± SEM. Statistical significance was assessed using the Student's t-test; p value ≤ 0.05 was considered significant.

## Results

### Basal and induced IGFBP-5 expression levels

In contrast to MMP-13 levels, which are significantly up-regulated in human OA cartilage [5,8], very little is known about the regulation of IGFBP-5 in this tissue. We first determined if IGFBP-5 was expressed in human chondrocytes and compared its level between normal and OA. Further, we looked at potential regulators of IGFBP-5 expression in OA chondrocytes.

**Figure 1:**
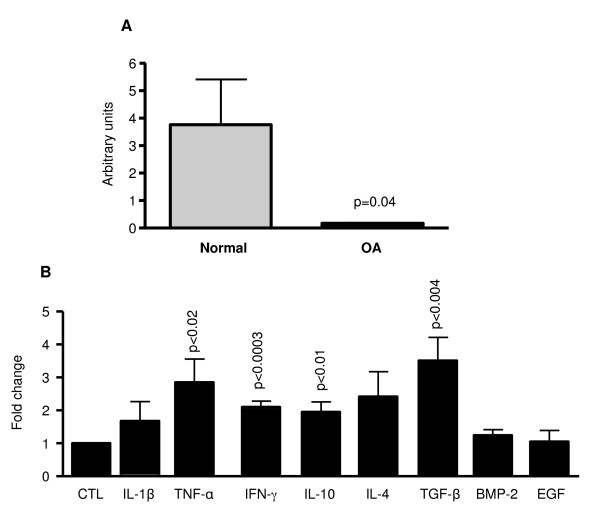
**IGFBP-5 expression and regulation in human chondrocytes**. **(A) **Total RNA was extracted from normal (n = 6) and OA (n = 6) human chondrocytes and processed for real-time PCR/SybrGreen. **(B) **Human OA chondrocytes (n = 5) were treated with cytokines and growth factors; total RNA was extracted and processed for real-time PCR/SybrGreen. Levels of the untreated (CTL) cells were given an arbitrary value of 1.

IGFBP-5 was expressed in normal and OA chondrocytes, and its level was significantly reduced (p < 0.04) in OA when compared to normal (Figure 1A). Treatment of OA chondrocytes with cytokines (IL-1β, TNF-α, IFN-γ, IL-10, and IL-4) and growth factors (TGF-β, BMP-2, and EGF) involved in arthritis pathophysiology showed that IGFBP-5 expression was increased by all the cytokines tested with statistical significance reached for TNF-α (p < 0.02), IFN-γ (p < 0.0003), and IL-10 (p < 0.01). TGF-β, but not the other two growth factors, BMP-2 and EGF, significantly up-regulated (p < 0.004) its expression level (Figure 1B).

### Bioinformatic prediction of miRNAs targeting MMP-13 andIGFBP-5 mRNAs

**Figure 2:**
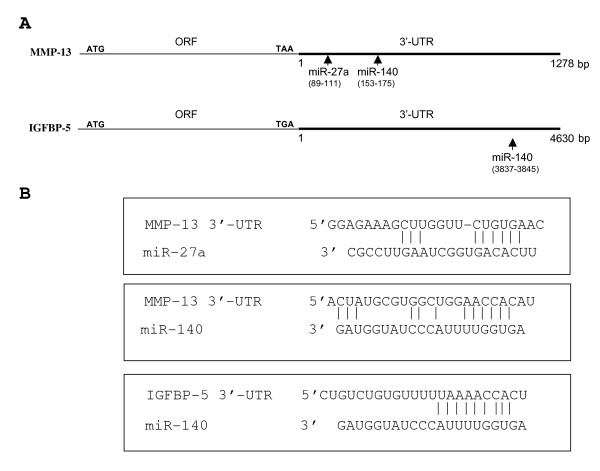
**Predicted recognition sequences for miR-140 and miR-27a in the 3'-UTR of the MMP-13 and IGFBP-5 mRNAs**. The 3'-UTRs were analyzed by computational programs to predict the presence of functional miR-140 and miR-27a sites. **(A) **Schematic representation of the mRNAs; ORF (open reading frame), ATG (start codon), TAA and TGA (stop codons), bp (base pair). The numbering starts at the first base following the stop codon. The arrows indicate the location of the potential pairing sites for the miRNAs. **(B) **Sequences of the potential pairing sites for miR-27a and miR-140. The bars indicate base pair homology.

To pursue the study of factors regulating MMP-13 and IGFBP-5 expression, more specifically those acting at the 3'-UTR, we investigated the role of miRNAs. To this end, the 3'-UTR sequences of MMP-13 and IGFBP-5 were analyzed by several computational algorithms (computational programs: http://www.microrna.org, http://www.targetscan.org, http://pictar.mdc-berlin.de, http://cbio.mskcc.org and http://lgmb.fmrp.usp.br/mirnapath) which utilize distinct parameters to predict the probability of a functional miRNA binding site within the 3'-UTR sequence of a given mRNA. All five computational programs predicted potential pairing sites for miR-140 and miR-27a in MMP-13 and IGFBP-5 3'-UTRs (Figure 2).

### Presence and effect of miR-140 and miR-27a in OA chondrocytes

Firstly, we investigated if miR-140 and miR-27a are expressed in human chondrocytes. Data revealed that both miRNAs are expressed in human OA chondrocytes at about the same level as the RNU24 control gene, which was given an arbitrary value of 1. Values of 0.88 and 0.94 fold change were recorded for miR-140 and miR-27a respectively. Prediction of a hybridization site does not necessarily confirm its functionality, thus validation is required. To this end, we used pre-miRNA and anti-miRNA molecules to determine the effects of miR-140 and miR-27a on the expression of target genes. Pre-miRNAs are small chemically modified double-stranded RNA molecules designed to mimic specific endogenous mature miRNAs, whereas anti-miRNAs are chemically modified nucleic acids designed to bind to and inhibit specific endogenous miRNAs. Therefore, pre-miRNAs enhance the inhibitory effect of miRNAs while anti-miRNAs antagonize the miRNA effect and cause increased expression of the target genes.

**Figure 3:**
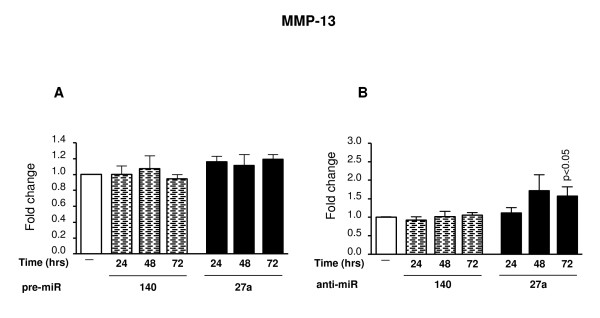
**Effect of pre- and anti-miR-140 and miR-27a on MMP-13 gene expression**. OA chondrocytes (n = 8) were transfected with the pre-miR **(A) **and anti-miR **(B) **molecules and incubated for 24, 48 and 72 hours. Total RNA was extracted and processed for real-time PCR/TaqMan. Levels from untreated chondrocytes (-) were assigned an arbitrary value of 1.

OA chondrocytes were transiently transfected with pre- or anti-miRNAs specific for miR-140 and miR-27a and incubated for 24, 48 and 72 hours (gene expression) and 72 hours (protein production). IGFBP-5 and MMP-13 production were determined. For comparison purposes, we also determined the expression of two other genes, IL-10 and bFGF, predicted as targets for miR-140 (bFGF) and miR-27a (IL10); these predictions were also obtained by the same five computational programs as described above. The results as illustrated in Figure 3 showed that treatment with pre-miR-140 or miR27a (Figure 3A) did not significantly affect MMP-13 expression levels, while transfection with anti-miR-27a (Figure 3B) increased MMP-13 expression with time, reaching statistical significance (p < 0.05) at 72 hours. Treatment with anti-miR-140 did not affect MMP-13 expression (Figure 3B).

**Figure 4:**
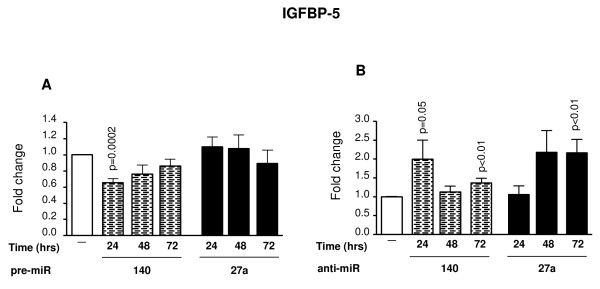
**Effect of pre- and anti-miR-140 and -27a on IGFBP-5 gene expression levels**. OA chondrocytes (n = 7-8) were transfected with the pre-miR **(A) **and anti-miR **(B) **molecules and incubated for 24, 48 and 72 hours. Total RNA was extracted and processed for real-time PCR/TaqMan. Levels from untreated chondrocytes (-) were assigned an arbitrary value of 1.

In contrast to MMP-13, these miRNAs differently affected the expression level of IGFBP-5 (Figure 4). Treatment with pre-miR-140 significantly inhibited (p = 0.0002) IGFBP-5 expression at as early as 24 hours (Figure 4A), while treatment with the anti-miR-140 significantly increased (p = 0.05) IGFBP-5 expression at 24 hours and 72 hours (p < 0.01) (Figure 4B). Because the cells were affected as early as 24 hours post-treatment, these data suggest that IGFBP-5 is a direct target of miR-140. IGFBP-5 expression, like that of MMP-13, was gradually affected by the anti-miR-27a; an increase was seen after 48 hours and significance (p < 0.01) reached after 72 hours of incubation (Figure 4B). The expression levels of IL-10 and bFGF were not affected by either pre- or anti-miRNAs (data not shown).

MMP-13 protein production followed the same pattern as the RNA expression profile. A significant increase was noted in chondrocytes treated with anti-miR-27a (1.5 ± 0.2 fold increase, p < 0.05, n = 8), but treatment with anti-miR-140 or with the pre-miRNAs did not significantly affect MMP-13 production. We also looked at the IGFBP-5 protein levels using the only commercially available ELISA. However, the IGFBP-5 basal level in four out of eight specimens was at the limit of detection of the assay. For the four specimens in which the IGFBP-5 level was detectable, data showed that treatment with both anti-miR-27a and anti-miR-140 induced a marked increase in level and values of 2.0 ± 1.1 and 3.0 ± 1.0 fold increase respectively were recorded.

All together, these data suggest that miR-140 acts directly on IGFBP-5 and miR-27a acts indirectly on both genes. Changes in the protein levels could result from the miRNAs promoting mRNA degradation combined with their interference with protein translation.

### Regulation of miR-140 and miR-27a expression levels inchondrocytes

**Figure 5:**
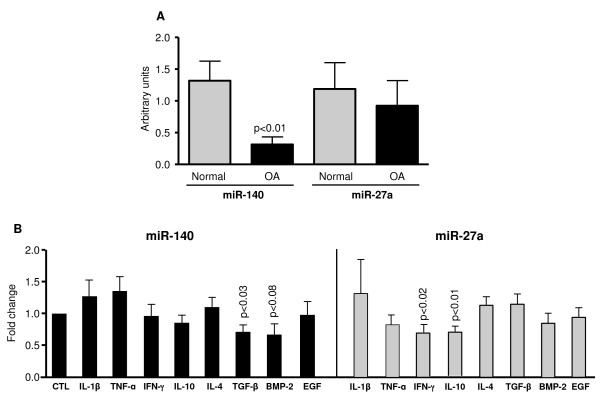
**Expression and regulation of miR-27a and miR-140 levels in human chondrocytes**. **(A) **Total RNA was extracted from normal (n = 6) and OA (n = 6) human chondrocytes and processed for real-time PCR/TaqMan. **(B) **OA chondrocytes (n = 5) were treated with cytokines and growth factors and miRNAs were extracted and processed for real-time PCR/TaqMan. Levels of the untreated (CTL) cells were given an arbitrary value of 1.

To determine whether miR-140 and miR-27a could play a role in the OA disease process, we compared their expression levels between normal and OA chondrocytes and identified possible regulatory factors. Data (Figure 5A) showed no significant difference in miR-27a expression between normal and OA chondrocytes although a slight decrease (23%) was observed in the OA. In contrast, miR-140 expression was significantly reduced (p < 0.01) in OA chondrocytes; a 77% reduction was found when compared to the expression in the normal cells.

OA chondrocytes were treated with cytokines and growth factors to identify those responsible for the differential expression of the miRNAs. miR-140 expression was significantly reduced (p < 0.03) by TGF-β (Figure 5B); it was also reduced by BMP-2, although not quite reaching statistical significance. None of the other factors tested affected miR-140 expression. In contrast, the cytokines IL-10 (p < 0.01) and IFN-γ (p < 0.02) significantly reduced the miR-27a levels.

## Discussion

The objective of this study was to complement the data on MMP-13 and IGFBP-5 regulation at the gene expression level by determining if miRNAs could affect the regulation of these genes and, if so, to identify and validate those miRNAs. Understanding the regulation of these factors is of great importance and could provide a new basis for the rationalization of a therapeutic strategy. Since several reports on miRNA profiling human cartilage [32], cancer [23] and general human tissues [21,36] have already been published, we chose to follow up on MMP-13 and IGFBP-5 and focus our research on the expression and regulation of miR-140 and miR-27a, as these miRNAs were identified with high prediction by the five computational programs used as possible regulators of both MMP-13 and IGFBP-5 expression.

Many factors contribute to the overall degradation of cartilage in OA. MMP-13 is well known to be up-regulated and to play a major role in the pathophysiological process of OA [1,4,5]. On the other hand, the exact function of IGFBP-5 in cartilage is not totally understood, but it is suggested to play a role as facilitator of IGF-1 availability in the tissue. Indeed, IGFBP-5 has been shown to associate with extracellular matrix macromolecules where it is protected from degradation and acts as a local reservoir for IGF-1 [11]. In this bound state, its affinity for IGF-1 is decreased when compared to the soluble state [11], indicating that IGFBP-5 would facilitate the delivery of this growth factor to its specific cell surface receptors. In OA, decreased levels of IGFBP-5 would diminish the capacity of the extracellular matrix to act as a reservoir for IGF-1; the free IGF-1 could then be sequestered by other IGFBPs, such as IGFBP-3 known to be increased in OA [37], resulting in its reduced bio-availability.

Data showed that the IGFBP-5 expression level was significantly decreased in human OA chondrocytes. This concurs with results from a study on another articular cell, the human subchondral bone osteoblast, in which the IGFBP-5 was down-regulated in OA [38]. However, it is in contrast to earlier studies reporting increased expression/production of IGFBP-5 in OA cartilage/chondrocytes [39,40]. Previous data [41], including our own [37], have shown that the IGFBP-5 protein basal level in human OA chondrocytes was undetectable or very low and subjected to the action of proteases. It is possible that variations may have occurred depending on the culture conditions as well as the variability of the different measurement methods used in those studies (immunohistochemistry, Western blot, semi-quantitative PCR). Here, we used more sensitive methods including quantitative PCR and a specific ELISA.

As IGFBP-5 is essential for maintaining IGF-1 anabolic activity, knowing the factor(s) responsible for its decreased expression level is important. Findings in the literature indicate that IGFBP-5 protein could be degraded by proteases and the serine protease Complement 1s, an enzyme present in the OA joint, was recently identified as being responsible for the cleavage of IGFBP-5 [41]. However, there are very few reports on gene expression. The present study showed that the IGFBP-5 gene expression is down-regulated by miR-140. This appears to be a direct effect, as IGFBP-5 is regulated as early as 24 hours post-treatment by the pre- and anti-miR-140. Although data showed that miR-140 is a regulatory factor of IGFBP-5, this does not imply that it is the only factor to down-regulate IGFBP-5, as miR-140 is also decreased in OA. Indeed, each gene is regulated by a variety of factors, some stimulatory and others suppressive. The decreased expression of IGFBP-5 in OA is the outcome of the interplay between these factors in which miR-140 plays a role.

Interestingly, MMP-13 and bFGF, which were also predicted to be miR-140 targets, were not affected by this miRNA. These latter data indicate that corroboration is necessary to conclude the specificity of a predicted target for a given miRNA.

Recent studies reported the role of some miRNAs in MMP regulation. For example, Stanczyk et al [24] demonstrated that the over-expression of miR-155 in RA synovial fibroblasts induced the repression of MMP-3 but not of MMP-13. However, MMP-3 has not yet been validated as a direct target of miR-155. On the other hand, Jones et al [42] recently reported that miR-9 could modulate MMP-13 expression, and Yamasaki et al [28] found, in OA cartilage, an association between the decreased expression of miR-146a and the increased MMP-13 expression level. Again, MMP-13 as a direct target of these miRNAs was not validated. Indirect regulation of MMP activity by miRNAs has also been recently reported in cancer cells [43]; miR-21 is over-expressed in glioblastomas and targets tissue inhibitor metalloprotease-3, a regulator of some MMPs. Moreover, on chondrocytes, miR-22 was shown to act on MMP-13 but through an effect on two other factors, PPARα and BMP-7 [32]. Thus, the control of gene expression by miRNAs can be both direct and indirect.

In this study, we show that MMP-13, as well as IGFBP-5, are likely indirect targets of miR-27a. Pre-miR-27a did not affect expression and anti-miR-27a treatment started to up-regulate transcription at 48 hours post-treatment, an increase which became significant after 72 hours. Of note, another gene predicted to be a target of miR-27a, IL-10, was not affected by either this pre- or anti-miRNA.

Data on MMP-13 and IGFBP-5 indicate that miR-27a affects the expression of another factor (or factors), which in turn acts on these two genes. It is likely that the factor is a stimulatory regulator of both IGFBP-5 and MMP-13 expression as they are affected only by the anti-miR-27a and not by the pre-miR-27a. The anti-miRNA would antagonize the inhibitory effect of miR-27a on the stimulatory factor resulting in its increased expression, which, in turn, would affect IGFBP-5 and MMP-13.

Although the identification of the miR-27a-targeted intermediate factor is currently ongoing, the computational programs have identified only a few miR-27a target genes that could have the potential to code for MMP-13 regulatory factors, and include PPARγ and Smad2. However, as the activation of PPARγ inhibits rather than stimulates MMP-13 expression [44], Smad2 is a more likely candidate. Although the IGFBP-5 promoter has been cloned and sequenced [45,46] it has not been fully characterized. However, our results show that TGF-β strongly stimulates IGFBP-5 expression, and Smad2 is implicated in TGF-β signaling [47] TGF-β has also been reported to up-regulate MMP-13 expression [8,48] and data further showed that the TGF-β-induced MMP-13 production in human OA chondrocytes was triggered by Smad proteins [49]. However, given the large number of potential miR-27a targets, the possibility that miR-27a targets two different regulatory factors for MMP-13 and IGFBP-5 is also considered.

Even though stimulators of IGFBP-5 were found in this study and include the cytokines TNF-α, IFN-γ and IL-10, and the growth factor TGF-β, they do not seem to be sufficient to maintain normal IGFBP-5 levels in OA chondrocytes, as the level of IGFBP-5 was significantly reduced in the diseased cells. This could be explained by the fact that OA chondrocytes do not produce these cytokines at high levels [50], in addition to the slightly increased miR-140 expression following TNF-α treatment. However, because of the differential role of TGF-β in the regulation of IGFBP-5 and miR-140, the low level of IGFBP-5 in OA chondrocytes was surprising. TGF-β significantly reduced miR-140 expression levels at the same time as strongly up-regulating IGFBP-5. Thus, TGF-β would act in two different ways to increase IGFBP-5: directly on its transcription, possibly at the promoter level, and indirectly by decreasing miR-140, a transcription inhibitor.

Reports on miRNA regulation/modulation are scarce. Our study is the first to show the regulation of the two miRNAs, miR-140 and miR-27a, in OA chondrocytes. Data show that TGF-β, known to be increased in OA cartilage [51,52], is a candidate for the reduced expression of miR-140 in these cells. As for miR-27a, among the factors tested, only IFN-γ and IL-10 decreased its expression. However, as mentioned above, these two factors are not found at high levels in OA [50] which could explain that only a slight reduction in miR-27a was found in OA chondrocytes.

## Conclusion

This study is the first to report on the regulation of miRNAs in OA chondrocytes. We show that miR-140 levels are decreased in OA and that the decrease could be attributed to the growth factor TGF-β. Data also suggest that miR-140 could act directly on decreasing IGFBP-5 expression but that miR-27a indirectly decreases both MMP-13 and IGFBP-5. These findings add another level of complexity to the overall regulation of MMP-13 and IGFBP-5, two factors involved in the OA pathological process. Data acquired from the study of these two genes could be the first step in generating more comprehensive knowledge of the regulation of other MMPs and IGFBPs involved in OA, which could open up novel avenues in therapeutic strategies targeting this disease.

## Competing interests

The authors declare that they have no competing interests.

## Authors' contributions

GT conceived of the study, was responsible for its design and coordination of the data acquisition and analysis, interpretation of the data, and wrote the manuscript. DH participated in the acquisition of the data and the statistical analysis. JPP participated in the analysis and interpretation of the data, as well as the manuscript preparation. ND contributed to the acquisition and validation of the study specimens. JMP participated in the study design, data analysis and interpretation, and writing of the manuscript. All authors read and approved the final manuscript.

## Pre-publication history

The pre-publication history for this paper can be accessed here:

http://www.biomedcentral.com/1471-2474/10/148/prepub
